# Experiences with the Russian Army of the Caucasus in Northern Persia

**Published:** 1919

**Authors:** 


***Experiences with the Russian Army of the Caucasus in Northern Persia***. By Lieut. T. LYLE HAZLETT. *The Military Surgeon*, October, 1917.

The American Field Hospital attached to the Army of the Caucasus was located in the far off town of Knoy. This was quite typical of the East, picturesque of course, but the hygienic conditions there were simply dreadful. The first thing Americans had to do on arriving there was to provide the town with pure, or at least good, water, and oblige the natives to clean out of the streets all the filth that was constantly heaped up in them.

The army hospitals were out of town; they did not prove at first very comfortable, but later on improvements were devised in their arrangement. Most important questions had to be looked after : the water supply, the disposal of sewage, the destruction of flies — these proving most troublesome.

In separate buildings were established a cholera hospital, a quarantine camp, a typhus hospital, and a dispensary for the natives.

Malaria headed the list of the most prevalent disease. Among 1390 cases admitted to the hospital during 10 months, were 260 cases of malaria. Next followed recurrent fever : 249 cases. Salvarsan injected intravenously had good results in severe cases of recurrent fever. 177 cases of typhus fever were cared for, with a mortality of 12 per cent. Typhoid occurred only at first ; after all the troops had received preventive vaccination, not a single case of typhoid was dealt with any more. The darkness of the skin of the Arabs and Turks rendered the diagnosis of typhoid rather difficult, especially at first.

The dysentery was of the bacillary form with an occasional case of amebic.

49 cases of scurvy occurred in spring-time.

The troops in the neighborhood all received prophylactic doses of cholera serum and were given quinine regularly.

Among all the medical cases admitted to the hospital, there were only three of syphilis and one of gonorrhea, while in the dispensary, among 2000 cases, no syphilitic case and only four of gonorrhea were dealt with. This low rate seems quite characteristic of the wholesomeness of the Russian peasant.

Among the natives, the most frequent diseases were tuberculosis and trachoma.

The surgical cases presented no particularities. They consisted of the usual wounds, as treated in every military hospital.

